# Ovarian lipid metabolic alterations in polycystic ovary syndrome: insights from proton magnetic resonance spectroscopy

**DOI:** 10.3389/fmed.2025.1652954

**Published:** 2025-10-15

**Authors:** Ke-Ying Wang, Ting Yang, Yi-Fan Ding, An-Rong Zeng, Ying Li, Jin-Wei Qiang

**Affiliations:** Department of Radiology, Jinshan Hospital, Fudan University, Shanghai, China

**Keywords:** polycystic ovary syndrome, lipid metabolism, magnetic resonance spectroscopy, fatty acid metabolism, lipid biomarkers

## Abstract

**Objectives:**

Altered ovarian lipid metabolism plays a critical role in the pathophysiology of polycystic ovary syndrome (PCOS). This study aimed to evaluate and compare ovarian lipid metabolic profiles in PCOS patients and healthy controls using proton magnetic resonance spectroscopy (^1^H-MRS).

**Method:**

This was a single-center prospective study. Single-voxel ^1^H-MRS was used to identify the lipid metabolism in 50 PCOS patients and 40 healthy controls (training cohort). A total of 34 PCOS patients and 39 controls underwent ^1^H-MRS on the contralateral ovaries (test cohort). Key lipid metabolites were identified and quantified using LCModel software. A combination of these key lipids using the binary logistic regression analysis was constructed to enhance the discrimination efficiency between PCOS patients and the controls.

**Results:**

Significant elevations were observed in lipid metabolites MM09 + Lip09, MM14 + Lip13a + Lip13b + MM12, MM21 + Lip21, Lip23, and Lip28 (e.g., MM09, macromolecule signal at 0.9 ppm; Lip09, lipid signal at 0.9 ppm) in PCOS patients compared to controls. The combination model of key lipids achieved an AUC of 0.89 [95% confidence interval (CI): 0.83–0.95], with sensitivity, specificity, positive predictive value (PPV) and negative predictive value (NPV) of 0.76, 0.95, 0.95, and 0.76, respectively, in the training cohort, and 0.88 (95% CI, 0.80–0.95), 0.76, 0.87, 0.84, and 0.81, respectively, in the test cohort. Pathway enrichment analysis revealed significant involvement of fatty acid metabolism, phospholipid synthesis, lipid signaling, and membrane organization pathways.

**Conclusion:**

The study highlights significant lipid metabolic disruptions in PCOS. Lip23 and Lip28 emerged as potential biomarkers for PCOS diagnosis. These findings enhance the understanding of PCOS pathophysiology and provide a foundation for future targeted therapeutic approaches.

## Introduction

Polycystic ovary syndrome (PCOS) is a common metabolic disorder affecting 5–20% of women of reproductive age (1, 2). Clinically, PCOS is characterized by menstrual irregularities, hirsutism, obesity, hyperinsulinemia, and hyperandrogenism (3). PCOS is also associated with long-term complications such as diabetes, cardiovascular diseases, and endometrial cancer (4).

The pathology of PCOS, believed to be complex and influenced by genetic, metabolic, and microenvironmental factors, remains unclear (5). A previous study has shown that the metabolic microenvironment of the ovaries plays a critical role in PCOS (6). Dyslipidemia is associated with abdominal obesity and hyperandrogenism in PCOS (7). Similarly, targeted metabolomics of the lipid profile in PCOS women showed elevated triglyceride levels, particularly those containing unsaturated fatty acids (8). Lipid metabolic analysis of follicular fluid from PCOS patients further revealed altered lipid metabolism in the ovaries (9). These findings suggest that lipid metabolism plays a crucial role in the metabolic dysfunction associated with PCOS.

Proton magnetic resonance spectroscopy (^1^H-MRS) is a non-invasive technique that detects metabolic changes in living tissues, allowing for the quantification of metabolite concentrations and biochemical alterations (10). Recent studies using *ex vivo* MRS and metabolomics have provided critical insights into amino acid and lipid dysregulation in the serum of PCOS patients (11). Therefore, it is possible to investigate the clinical usefulness of MRS for lipid metabolism in PCOS patients.

We believe that non-invasive *in vivo* ^1^H-MRS may provide valuable information for the clinical diagnosis of PCOS and help further elucidate the mechanisms of ovarian lipid dysfunction in these patients. This study aims to utilize ^1^H-MRS to analyze and compare ovarian lipid metabolic profiles in PCOS patients and healthy controls, and to identify the key lipids and lipid metabolic pathways disrupted in PCOS.

## Materials and methods

### Ethics

This prospective study was approved by the Review Board of the local hospital (Approval number: JIEC-2019-S31), and written informed consent was obtained from all participants. Patients or the public were not involved in the design, conduct, reporting, or dissemination plans of this research.

### Study design and general information

The study included 50 consecutive patients from the Gynecological Endocrinology outpatient clinic and 40 healthy volunteers from the Health Management Center between December 2019 and December 2020. Clinical data, laboratory results, and data were collected from both groups. All subjects underwent MRS scanning on at least one ovary, with data from these scans used as the training cohort. A subset of subjects successfully underwent MRS scanning on their other ovary as well, with the data from these scans used as the test cohort. The study aimed to analyze and compare quantitative lipid indices to investigate ovarian lipid metabolic changes between PCOS patients and healthy controls.

The inclusion criteria for the PCOS group were as follows: meeting two of the following three criteria (12): (1) menstrual irregularities, defined as menstrual cycles shorter than 21 days or longer than 35 days within 1–3 years after menarche; menstrual cycles shorter than 21 days or longer than 35 days more than 3 years after menarche; fewer than eight menstrual cycles in a year; any menstrual cycle lasting more than 90 days 1 year after menarche; or absence of menstruation after the age of 15 years or within 3 years of breast development; (2) clinical or biochemical hyperandrogenism, with clinical hyperandrogenism presenting as acne, hirsutism, or alopecia. Hirsutism was diagnosed with a Ferriman–Gallwey score of >4–6, and alopecia was assessed using the Ludwig Visual Score. Biochemical hyperandrogenism was defined as a free testosterone level >63 ng/dL (2.8 nmol/L); (3) polycystic ovaries, indicated by ultrasound showing 12 or more small follicles (2–9 mm in diameter) arranged in a circular or scattered pattern in one ovary and/or an ovary volume (OV) >10 cm^3^. The control group consisted of healthy women of reproductive age. All participants were between menarche and menopause, aged 20 to 31 years. None were pregnant or on any medication at the time of examination.

The exclusion criteria were as follows: (1) ovulatory dysfunction due to other diseases, such as thyroid dysfunction or hyperprolactinemia; or hyperandrogenism due to adrenal hyperplasia, severe insulin resistance syndrome, or androgen-secreting tumors; (2) idiopathic hyperandrogenism or hirsutism; (3) presence of a dominant follicle, cyst, or corpus luteum.

### Clinical data

Clinical information was recorded for each participant, including age, menstrual status, physical examination results, and body mass index (BMI = weight/height^2^). After fasting for 4–6 h prior to the test, approximately 10 mL of venous blood was collected for laboratory testing, including free testosterone (T), luteinizing hormone (LH), and follicle-stimulating hormone (FSH). For controls and PCOS patients with regular menstrual cycles, blood samples were taken during the follicular phase, specifically between days 3 and 5 of the menstrual cycle; for PCOS patients with irregular cycles, blood was drawn at any time.

### MRI and MRS examination

A 3.0-T MRI scanner (MAGNETON Verio, Siemens, Erlangen, Germany) was used with a body coil as the transmitter and a pelvic phased-array coil as the receiver. For all subjects, blood samples and MRS scans were conducted on the same day. During the scan, patients were positioned supine and instructed to breathe calmly. The scanning range extended from the anterior superior iliac spine to the upper segment of the femur. Routine MRI sequences were performed, including axial, coronal, and sagittal T2-weighted imaging (T2WI). Single-voxel ^1^H-MRS was performed with the voxel (8 × 8 × 8 mm) positioned within the largest visible follicular structure on T2-weighted images, avoiding the ovarian stroma and adjacent tissues. The point-resolved spectroscopy (PRESS) pulse sequence was used for the ^1^H-MRS. A water reference scan was acquired for each voxel prior to lipid acquisition using the same voxel placement and acquisition parameters. The water signal served as an internal concentration reference for absolute lipid quantification, ensuring consistency across all subjects. B0 shimming was performed prior to spectral acquisition to minimize magnetic field inhomogeneities within the voxel of interest (VOI). A first-order automatic shimming technique was used to optimize the homogeneity of the magnetic field. The water linewidth within the VOIs was measured after shimming and ranged from 10 to 15 Hz. Sequence and parameter details are listed in Supplementary Table 1.

### MRS data processing

The raw spectral data were imported into LCModel software for quantification, where absolute and relative concentrations of lipids were calculated by fitting *in vivo* spectra to simulated basis sets of known lipids. Simulated basis sets were standard for LCModel, with validation performed by comparing key lipid peaks with those reported in previous ovarian MRS studies. The spectral range analyzed spanned 0.2 to 4.2 ppm. The accuracy of the lipid quantitation was assessed using Cramer–Rao lower bounds (CRLBs), a measure of the uncertainty in the estimated concentration. Only lipids detected in at least 80% of the spectra, with a signal-to-noise ratio (SNR) of >10 and a CRLB value of <20%, were considered reliable and selected for further analysis. Lipid concentrations were normalized to the unsuppressed water peak signal to account for voxel volume and coil sensitivity, ensuring consistent quantification.

### Key lipid identification

Absolute lipid concentrations were quantified in millimolar (mM) units. Absolute quantification was performed by normalizing the lipid signals to water peak intensity, taking into account factors of voxel size, field strength, and coil sensitivity. Principal component analysis (PCA) was used to reduce the dimensionality of the lipid data, highlighting major patterns and separating groups based on their lipid profiles, which enabled the identification of broad trends and initial group clustering without requiring prior assumptions. Partial least squares discriminant analysis (PLS-DA) was applied to enhance group separation and identify metabolites with the greatest discriminatory power. Key metabolites were selected based on their statistical significance (*p* < 0.05) and a variable importance in projection (VIP) score of >1, ensuring robustness across both the training and test cohorts.

### Discrimination of key lipids and validation

The AUCs of the key lipids in differentiating PCOS from controls were calculated. A combined model (a combination of the key lipids) was constructed using binary logistic regression to enhance the discrimination efficiency between PCOS and controls. The predictive performances of the combined model were also evaluated.

### Metabolic enrichment and pathway analysis

The LIPID MAPS database^1^ was used for key lipid classification. The lipid categories were confirmed based on statistical significance for the identified key lipids. The KEGG^2^ and Reactome^3^ databases were used for pathway analysis. Relevant biological pathways were identified by considering both direct and indirect lipid interactions associated with ovarian lipid metabolism in PCOS.

### Statistical analysis

Statistical analysis was performed using R software (Version 4.4.0, https://www.r-project.org/). Normally distributed quantitative parameters were compared using independent *t*-tests, while non-normally distributed parameters were compared using the Mann–Whitney *U*-test. Qualitative parameters were assessed using the chi-squared test. The receiver operating characteristic (ROC) curves were used to evaluate the diagnostic efficiency of these parameters. The combination of the parameters (key lipids) was performed using a linear combination. Fisher’s exact test was used for enrichment analysis. Data were presented as mean ± SD. A *p*-value of <0.05 was considered statistically significant.

### Sample size determination

To ensure statistical validity, we performed a *post-hoc* power analysis based on the effect size of Lip23 (which showed the highest AUC in the training cohort). Using a two-sided *α* = 0.05, a power of 80%, and the observed group mean difference and standard deviation, we determined that a minimum of 34 participants per group would be sufficient to detect a statistically significant difference.

## Results

### Clinical characteristics

**Figure 1:**
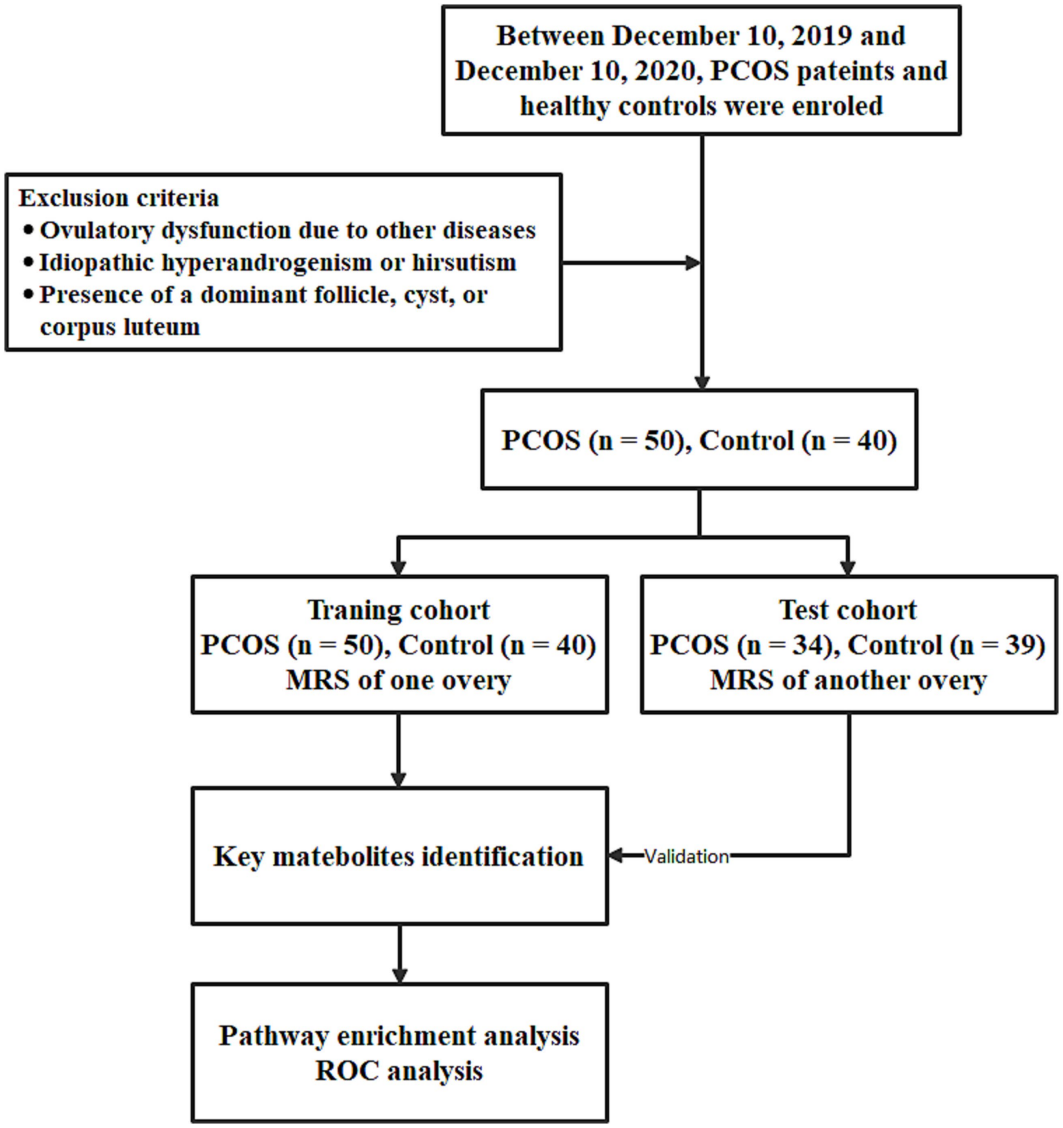
Workflow of this study. PCOS, polycystic ovary syndrome, MRS, magnetic resonance spectroscopy, and ROC, receiver operating characteristic.

The workflow of this study is shown in Figure 1. The clinical characteristics of PCOS patients and controls in both the training and test cohorts are shown in Table 1. PCOS patients had a significantly higher BMI than controls (training: *p* < 0.001; test: *p* < 0.001). OV was also notably larger in PCOS patients (training: *p* < 0.001; test: *p* < 0.001), and they exhibited a significantly higher follicle count (training: *p* < 0.001; test: *p* < 0.001).

**Table 1:** Comparison of baseline clinical characteristics and hormonal levels between PCOS patients and control groups in the training and test cohorts.

Parameters	Training cohort			Test cohort		
	Control (*N* = 40)	PCOS (*N* = 50)	*p*-value	Control (*N* = 39)	PCOS (*N* = 34)	*p*-value
Age (years)	26 ± 2.4	25 ± 5.0	0.29	25 ± 2.5	24 ± 5.0	0.344
BMI	20 ± 2.4	24 ± 4.7	<0.001	20 ± 2.0	25 ± 4.0	<0.001
Overy volume (mm^3^)	6.9 ± 2.8	12.2 ± 4.6	<0.001	7.0 ± 2.8	12.1 ± 4.7	<0.001
Follicle count	10.0 ± 5.1	23.2 ± 9.1	<0.001	10.5 ± 4.1	24.3 ± 9.0	<0.001
LH (IU/L)	5.3 ± 3.3	12.6 ± 6.9	<0.001	5.4 ± 3.6	13.2 ± 7.3	<0.001
FSH (IU/L)	8.2 ± 1.6	6.3 ± 1.7	<0.001	8.3 ± 1.6	6.0 ± 1.8	<0.001
LH/FSH	0.6 ± 0.5	2.0 ± 1.1	<0.001	0.6 ± 0.5	2.3 ± 1.2	<0.001
Testosterone (nmol/L)	0.5 ± 0.16	0.8 ± 0.50	<0.001	0.5 ± 0.15	0.9 ± 0.40	<0.001
Hyperandrogenism			<0.001			<0.001
Negative	31 (77.5%)	10 (20.0%)		31 (79.5%)	4 (11.8%)	
Positive	9 (22.5%)	40 (80.0%)		8 (20.5%)	30 (88.2%)	
Oligo anovulation			<0.001			<0.001
Negative	35 (87.5%)	2 (4.0%)		34 (87.2%)	2 (5.9%)	
Positive	5 (12.5%)	48 (96.0%)		5 (12.8%)	32 (94.1%)	

PCOS, polycystic ovary syndrome; BMI, body mass index; LH, luteinizing hormone; FSH, follicle-stimulating hormone.

Significant hormonal differences were noted between the two groups. PCOS patients had elevated LH levels (training: *p* < 0.001; test: *p* < 0.001) and reduced FSH levels (training: *p* < 0.001; test: *p* < 0.001), resulting in a significantly higher LH/FSH ratio in PCOS patients. T levels were also significantly higher in PCOS patients (training: *p* < 0.001; test: *p* < 0.001). Additionally, hyperandrogenism and oligo-anovulation were more prevalent in the PCOS group in both cohorts (both *p* < 0.001).

### Key lipid identification

**Figure 2:**
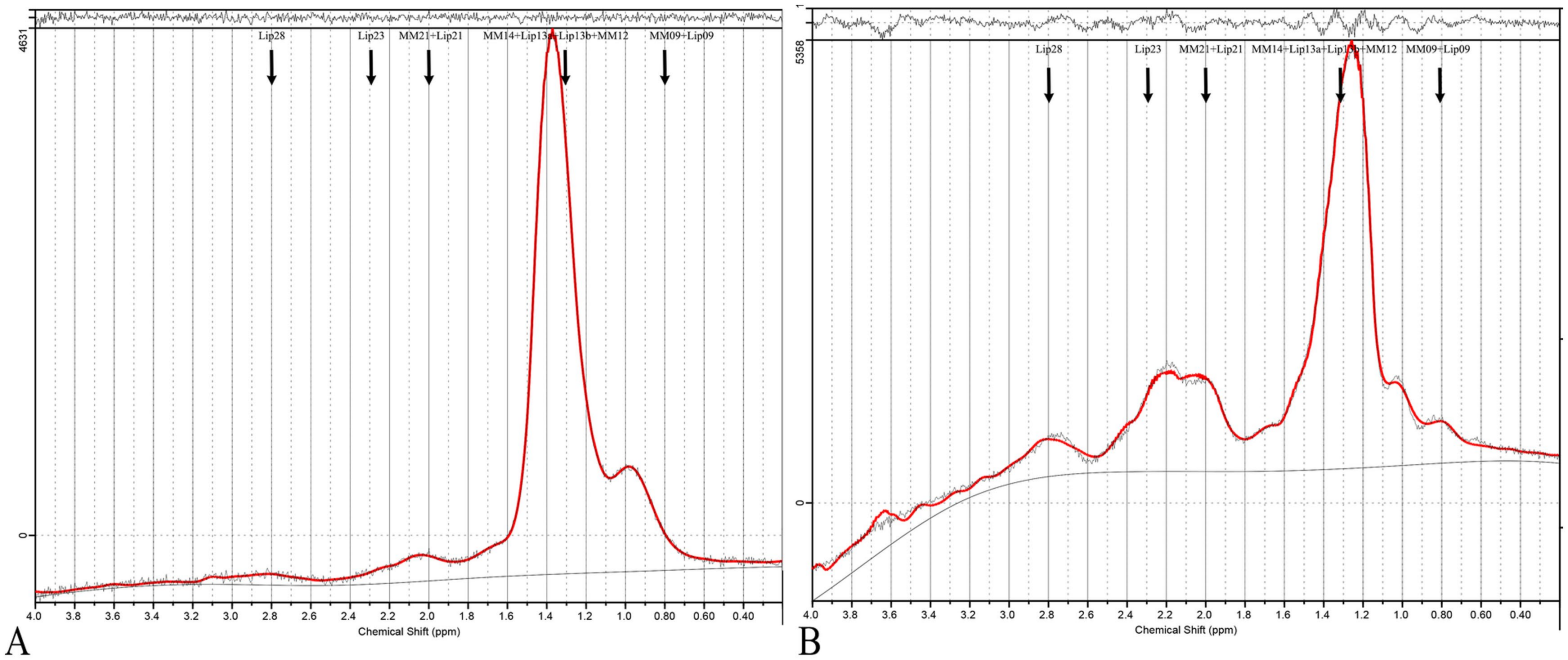
MRS of PCOS and control cases. **(A)** Shows a typical ^1^H-MRS spectrum of the ovary from a healthy control, while **(B)** displays the spectrum from a PCOS patient. The spectra are presented as plots of signal intensity against chemical shift (ppm). In the PCOS spectrum **(B)**, distinct peaks are labeled corresponding to key lipids: MM09 + Lip09, MM14 + Lip13a + Lip13b + MM12, MM21 + Lip21, Lip23, and Lip28. These lipids were significantly elevated in PCOS patients compared to controls.

**Figure 3:**
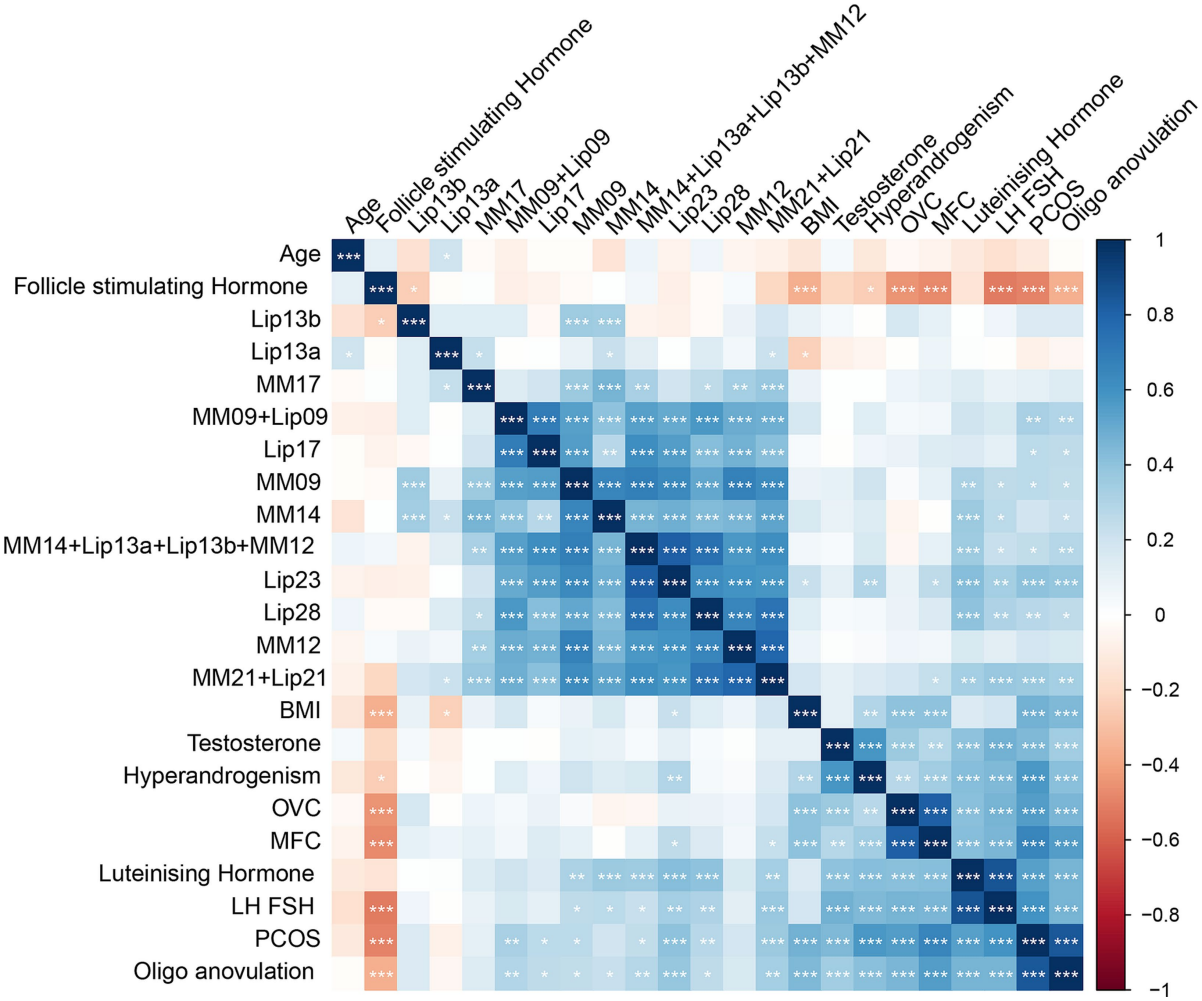
A correlation heatmap of metabolites and clinical parameters in PCOS and control groups. This heatmap illustrates the correlation matrix between key metabolites and clinical parameters in PCOS patients and controls. The color scale represents the correlation coefficients, where red indicates a positive correlation and blue indicates a negative correlation, with intensity corresponding to the strength of the correlation. The heatmap was generated using Pearson’s correlation analysis.

Using PCA followed by PLS-DA (Supplementary Figure 1), several key lipids were identified as significant markers of metabolic disruption in PCOS. Lipids meeting the criteria of *p* < 0.05 and a VIP score of >1 in both the training and test cohorts were considered key lipids. The identified key lipids included MM09 + Lip09, MM14 + Lip13a + Lip13b + MM12, MM21 + Lip21, Lip23, and Lip28 (e.g., MM09, macromolecule signal at 0.9 ppm; Lip09, lipid signal at 0.9 ppm). These lipids showed significant differences between PCOS patients and controls (Figure 2 and Supplementary Table 2). The correlation heatmap of the key lipids and clinical parameters is shown in Figure 3.

Standardized LIPID MAPS nomenclature was used to define the key lipid species. Specifically, each lipid resonance has been annotated with its corresponding chemical class and subclass based on its chemical shift and assignment in the LIPID MAPS database.^4^ Lip09 corresponds primarily to terminal methyl protons in fatty acyl chains (fatty acyls, FA01). Lip13 reflects methylene protons adjacent to double bonds (unsaturated fatty acids, FA12). Lip21 and Lip28 are associated with the bis-allylic and allylic methylene groups, primarily from polyunsaturated fatty acids (PUFAs, FA13/FA14). Lip23 is linked to choline-containing phospholipids (e.g., phosphatidylcholine, glycerophospholipids, and GP01).

### Discrimination of key lipids and validation

**Figure 4:**
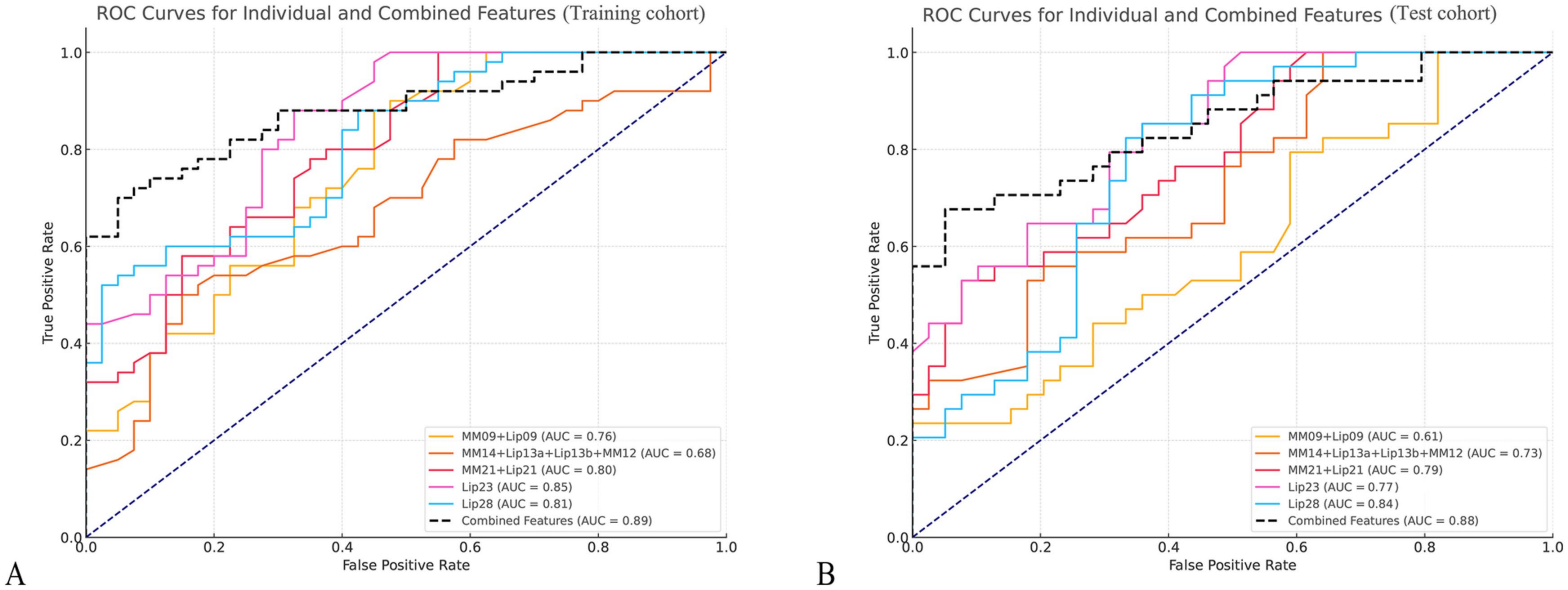
Receiver operating characteristic (ROC) curves for key metabolites in PCOS and control groups. **(A,B)** Display the ROC curves for six key metabolites in differentiating PCOS patients from healthy controls in the training cohort **(A)** and the test cohort **(B)**. The curves represent sensitivity (true positive rate) plotted against 1-specificity (false positive rate) for each metabolite.

For evaluating the discrimination ability of the identified key lipids between PCOS patients and controls, Lip23 demonstrated the highest power, with an AUC of 0.85 in the training cohort (*p* < 0.001) and of 0.77 in the test cohort (*p* = 0.001). Lip28 also exhibited strong discrimination ability, with an AUC of 0.81 in the training cohort (*p* = 0.009) and 0.84 in the test cohort (*p* = 0.001); and MM09 + Lip09, MM14 + Lip13a + Lip13b + MM12, and MM21 + Lip21 displayed moderate discrimination ability, with AUCs ranging from 0.61 to 0.80 across both cohorts (Figure 4 and Supplementary Table 2).

The combination model of key lipids reached an AUC of 0.89, with a 95% confidence interval (CI) of 0.83–0.95, and sensitivity, specificity, positive predictive value (PPV), and negative predictive value (NPV) of 0.76, 0.95, 0.95, and 0.76, respectively, in the training cohort; and of 0.88 (95% CI, 0.80–0.95), 0.76, 0.87, 0.84, and 0.81, respectively, in the test cohort (Figure 4).

### Subgroup analyses by BMI/PCOS phenotype

Subgroup analyses showed that overweight/obese PCOS patients (BMI ≥25) exhibited significantly higher levels of MM14 + Lip13a + Lip13b + MM12 compared to normal-weight PCOS patients (BMI <25), with *p*-values of 0.071and 0.044 in the training and test cohorts, respectively. Patients with the phenotype (HA + PCOM) showed higher levels of MM14 + Lip13a + Lip13b + MM12 (*p* = 0.081 and *p* = 0.003), Lip21 (*p* = 0.008 and *p* = 0.037), and Lip23 both in the training and test cohorts. Patients with the full phenotype (OA + PCOM) showed higher levels of Lip23 (*p* = 0.077 and *p* = 0.036) and Lip28 (*p* = 0.032 and *p* = 0.024), both in the training and test cohorts. Patients with the full phenotype (HA + OA) showed higher levels of MM14 + Lip13a + Lip13b + MM12 (*p* = 0.009 and *p* = 0.001) and Lip28 (*p* = 0.079 and *p* = 0.019), both in the training and test cohorts. Patients with the full phenotype (HA + OA + PCOM) showed higher levels of MM09 + Lip09 (mean 0.17, 95% CI: 0.08–0.25, *p* = 0.008 and mean 0.11, 95% CI: 0.04–0.19, *p* = 0.099), MM14 + Lip13a + Lip13b + MM12 (mean 0.14, 95% CI: 0.05–0.24, *p* = 0.051 and mean 0.19, 95% CI: 0.01–0.38, *p* = 0.001), and Lip28 (mean 0.20, 95% CI: 0.07–0.32, *p* = 0.040 and mean 0.23, 95% CI: 0.10–0.36, *p* = 0.060) both in the training and test cohorts.

### Enrichment and pathway analyses

**Figure 5:**
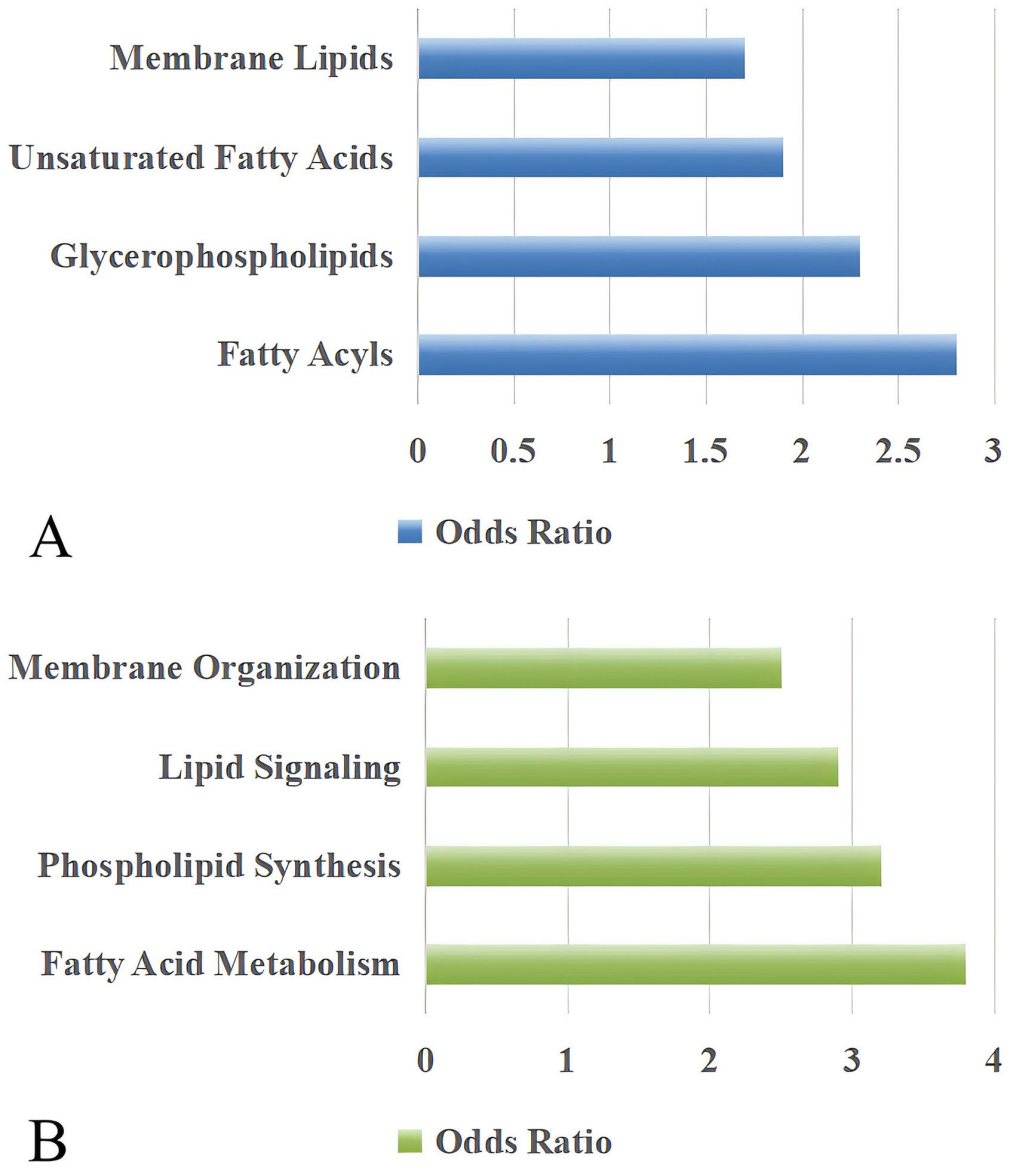
Pathway analysis of lipid metabolism and organization in PCOS patients. **(A)** Key lipid categories, including membrane lipids, unsaturated fatty acids, glycerophospholipids, and fatty acyls, are highlighted in PCOS-related pathways. **(B)** Biological processes associated with lipid metabolism, including membrane organization, lipid signaling, phospholipid synthesis, and fatty acid metabolism, show significant pathway involvement. The odds ratio reflects the relative likelihood of pathway enrichment compared to a reference group, with higher values indicating stronger pathway relevance.

Enrichment and pathway analyses revealed significant associations between the identified key lipids and disrupted metabolic pathways in PCOS (Figure 5). Enrichment analysis showed that fatty acyls (odds ratio 2.8, *p* = 0.001), glycerophospholipids (odds ratio 2.3, *p* = 0.003), fatty acids (odds ratio 1.9, *p* = 0.008), and membrane lipids (odds ratio 1.7, *p* = 0.012) were significantly enriched. Pathway analysis showed that fatty acid metabolism, phospholipid synthesis pathway, lipid signaling pathways, and membrane organization pathways were involved in the lipid metabolism in PCOS.

## Discussion

This study utilized ^1^H-MRS to assess ovarian lipid metabolic changes in PCOS patients compared to healthy controls. The results revealed significant elevations in the lipids of MM09 + Lip09, MM14 + Lip13a + Lip13b + MM12, MM21 + Lip21, Lip23, and Lip28 in PCOS patients, aligning with previous findings that highlight altered lipid metabolic pathways in PCOS, including fatty acyls, glycerophospholipids, fatty acids, and membrane lipid metabolism.

The identification of Lip09 and Lip13 in PCOS patients suggests enhanced fatty acid metabolism, particularly in pathways related to lipogenesis and beta-oxidation. Elevated Lip09 has been associated with increased lipid accumulation in tissues, reflecting disrupted lipid homeostasis and energy storage mechanisms in PCOS. Similarly, Lip13, which includes components such as monounsaturated and polyunsaturated fatty acids, indicates heightened activity in fatty acid synthesis and desaturation processes. These findings align with previous studies, which observed short-chain fatty acids in patients’ serum by mass spectrometry methods (13). Additionally, another study highlighted that the fatty acids of C14:0, C16:1, C18:1n-9C, C18:1n-7, and C20:3n-6 levels are elevated in women with PCOS (14). These observations suggest that Lip09 and Lip13 are integral markers of dysregulated lipid metabolism, reflecting the broader metabolic impairments in PCOS.

The identification of Lip21 and Lip28 in PCOS patients indicates significant alterations in unsaturated fatty acid metabolism, emphasizing the disruption of lipid pathways. Lip21, primarily associated with monounsaturated fatty acids, reflects enhanced *de novo* lipogenesis, which has been linked to insulin resistance and chronic inflammation (15). Similarly, Lip28, representing polyunsaturated fatty acids, suggests potential imbalances in omega-6 and omega-3 fatty acid pathways, which are known contributors to inflammatory responses (16, 17). Yang et al. (18) reported that omega-3 fatty acids may be recommended for the treatment of PCOS with insulin resistance. These findings collectively underscore that Lip21 and Lip28 serve as markers of lipid metabolic disruption and inflammatory processes in PCOS, offering insights into potential therapeutic targets for mitigating metabolic and reproductive dysfunction.

The detection of Lip23 in PCOS patients indicates active membrane lipid synthesis, a critical process for cellular structure and function. Lip23 is associated with the synthesis of glycerophospholipids and other structural lipids required for membrane biogenesis. This finding aligns with Mao et al. (19), who reported that hypermethylation of LPCAT1 and PCYT1A genes in PCOS patients led to the dysregulation of glycerophospholipid metabolism, including reduced phosphatidylcholine synthesis, a major component of cell membranes. Additionally, Leung et al. (20) identified increased expression of genes related to triacylglycerol synthesis in adipose stem cells of PCOS women, further emphasizing lipid pathway dysregulation. These studies collectively underscore the role of altered membrane lipid synthesis in PCOS pathology, which may contribute to the structural and functional changes observed in ovarian cells. Future research should explore the potential of Lip23 as a biomarker for disrupted lipid metabolism and its implications for PCOS-related metabolic dysfunction.

The elevated lipid levels, particularly Lip09, Lip13, Lip21, and Lip28, observed in the current study further underscore the potential role of insulin resistance in disrupting ovarian metabolism. Insulin resistance has been shown to impair the normal utilization of glucose, leading to an increased reliance on lipids and amino acids for energy production. This metabolic shift may explain the accumulation of lipids and amino acids in the ovarian microenvironment, which negatively impacts oocyte development and overall fertility outcomes in PCOS patients (21). These findings are also consistent with prior studies showing elevated circulating LDL, triglycerides, and long-chain fatty acids in insulin-resistant PCOS populations, suggesting systemic and local dysregulation of lipid handling (22). The accumulation of these lipids can impair ovarian function, disrupting folliculogenesis and contributing to infertility in PCOS patients.

### Strengths and limitations

The ovary’s small, mobile nature, particularly in premenopausal women, benefits from single-voxel MRS’s superior signal-to-noise ratio, robust water suppression, and enhanced spectral quality in confined tissue volumes. Single-voxel MRS’s shorter acquisition time reduces motion artifacts from respiration, bowel peristalsis, and patient movement, ensuring better spectral quality and participant compliance. Additionally, manual voxel placement within the largest visible follicle provided precise anatomical targeting and consistent acquisition across subjects, while CSI’s fixed grid structure would have limited our ability to target specific follicular regions and avoid contamination from adjacent bowel and uterine tissues. Despite the robust findings of this study, there are some limitations. First, the study did not account for different PCOS phenotypes (e.g., obesity, insulin resistance, or hyperandrogenism), which may have contributed to the variability in the results. Second, this study focused on metabolites within the 0.2–4.0 ppm range, which includes the most commonly assessed metabolites. The downfield region beyond 4.0 ppm, while less frequently analyzed, might provide insights into additional lipid metabolism in PCOS. Future studies could explore these regions to capture a broader range of lipid changes potentially relevant to PCOS pathophysiology. Third, the study was conducted at a single center with a limited sample size. Future multicenter studies with larger populations are needed to further validate these findings and explore potential therapeutic strategies targeting ovarian metabolism in PCOS patients. Fourth, ^1^H-MRS provides chemical shift information related to functional groups of lipids, but it does not allow for the precise identification of complete lipid structures or subclasses. Fifth, while *in vivo* ^1^H-MRS provides localized information specific to the ovarian microenvironment, future studies should integrate blood-based lipidomics to explore systemic correlations and evaluate the potential of these lipids as non-invasive biomarkers for PCOS. Furthermore, a notable limitation of this study is the inability of 1H-MRS to distinguish between lipid isomers, as the spectral resolution of *in vivo* MRS does not permit the precise separation of overlapping peaks from structurally similar lipid species.

## Conclusion

In conclusion, this study provides evidence of significant metabolic alterations in the ovaries of PCOS patients, particularly involving lipid metabolism. These findings enhance our understanding of PCOS pathophysiology and may aid in developing more targeted therapeutic approaches for managing the reproductive and lipid metabolic symptoms of PCOS.

## Data Availability

The raw data supporting the conclusions of this article will be made available by the authors, without undue reservation.

## Footnotes

^1^ https://www.lipidmaps.org/

^2^ https://www.genome.jp/kegg/

^3^ https://reactome.org/

^4^ https://www.lipidmaps.org/index.php
