# Supplementary material for: Ovarian lipid metabolic alterations in polycystic ovary syndrome: insights from proton magnetic resonance spectroscopy
Source: Front Med (Lausanne). 2025 Oct 15;12:1652954. doi: 10.3389/fmed.2025.1652954 (PMC12568718; doi:10.3389/fmed.2025.1652954)
Supplement: Supplementary file 1 [file Data_Sheet_1.docx]

Supplementary Table 1 MRI and ^1^H-MRS scan parameters

| Parameters | Axial T2WI | Sagittal T2WI | Coronal T2WI | ^1^H-MRS |
| --- | --- | --- | --- | --- |
| Sequence | SE | SE | SE | MRS |
| Repetition time (ms) | 5500 | 8460 | 1400 | 4700 |
| Echo time (ms) | 102 | 102 | 104 | 71 |
| Slice thickness (mm) | 2.0 | 2.0 | 5.0 | - |
| Slice gap (mm) | 0.6 | 0.6 | 1.0 | - |
| Field of view (mm) | 353 × 353 | 265 × 479 | 300 × 542 | - |
| Matrix | 320 × 320 | 320 × 320 | 320 × 320 | - |
| Acquisition time (s) | 106 | 84 | 216 | 433 |

Supplementary Table 2 Comparison of Metabolites in Absolute Values Between PCOS and Control Groups in the Training and Test Cohorts

|  | Train | | | | Test | | | |
| --- | --- | --- | --- | --- | --- | --- | --- | --- |
| Metabolite (Mm) | Fold Change | P-value | VIP Score | AUC | Fold Change | P-value | VIP Score | AUC |
| MM09+Lip09 (0.9 ppm) | 11.9 | 0.002 | 1.2 | 0.76 | 6.2 | 0.008 | 1.3 | 0.61 |
| MM09 (0.9 ppm) | 4.2 | 0.013 | 0.9 | 0.63 | 6.9 | 0.016 | 1.3 | 0.59 |
| MM12 (1.2 ppm) | 2.7 | 0.082 | 1.0 | 0.58 | 1.2 | 0.732 | 0.5 | 0.57 |
| MM14+Lip13a+Lip13b+MM12 (1.3 ppm) | 6.0 | 0.022 | 1.2 | 0.68 | 5.4 | 0.007 | 1.6 | 0.73 |
| Lip13a (1.3 ppm) | 0.8 | 0.483 | 0.6 | 0.55 | 1.7 | 0.201 | 0.8 | 0.51 |
| Lip13b (1.3 ppm) | 11.1 | 0.151 | 0.9 | 0.53 | 6.8 | 0.1 | 1.0 | 0.50 |
| MM14 (1.4 ppm) | 2.2 | 0.064 | 0.9 | 0.65 | 1.2 | 0.512 | 0.4 | 0.56 |
| Lip17 (1.7 ppm) | 14.7 | 0.013 | 1.2 | 0.59 | 11.3 | 0.001 | 1.9 | 0.79 |
| MM17 (1.7 ppm) | 1.5 | 0.268 | 0.6 | 0.62 | 1.1 | 0.776 | 0.2 | 0.51 |
| MM21+Lip21 (2.02 ppm) | 27.9 | <0.001 | 1.4 | 0.80 | 25.2 | <0.001 | 1.7 | 0.79 |
| Lip23 (2.23 ppm) | 51.5 | <0.001 | 2.0 | 0.85 | 76.0 | 0.001 | 1.4 | 0.77 |
| Lip28 (2.75 ppm) | 15.8 | 0.009 | 1.2 | 0.81 | 13.0 | 0.013 | 1.1 | 0.84 |

Lip09, lipid signal at 0.9 ppm (-CH3); Lip13a, lipid signal at 1.3 ppm (-CH2-)n; Lip13b, lipid signal at 1.3 ppm (-CH2-); Lip17, lipid signal at 1.7 ppm (-CH2-); Lip21, lipid signal at 2.02 ppm (-CH2-CH=CH-); Lip23, lipid signal at 2.23 ppm (-CH2-COO-); Lip28, lipid signal at 2.75 ppm (=CH-CH2-CH=); MM09, macromolecule signal at 0.9 ppm; MM12, macromolecule signal at 1.2 ppm; MM14, macromolecule signal at 1.4 ppm; MM17, macromolecule signal at 1.7 ppm; MM21, macromolecule signal at 2.02 ppm


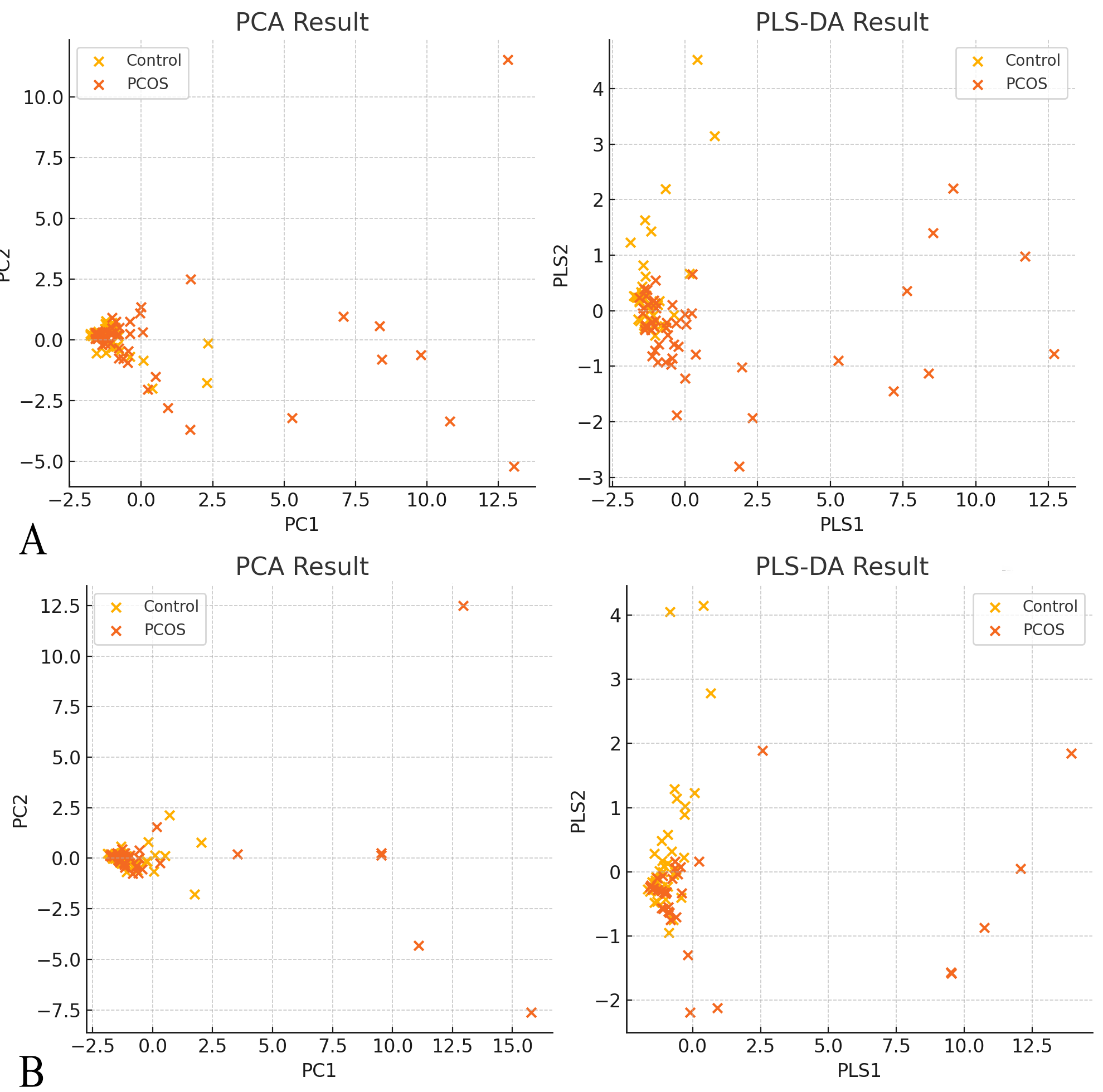


Supplementary Figure 1 PCA and PLS-DA analysis of metabolite profiles in PCOS and control groups. This figure shows the results of principal component analysis (PCA) and partial least squares discriminant analysis (PLS-DA) applied to the metabolomic data from PCOS patients and healthy controls in both the training cohort (A) and the test cohort (B).
